# The Role of the Ventromedial Prefrontal Cortex in Purchase Intent Among Older Adults

**DOI:** 10.3389/fnagi.2016.00189

**Published:** 2016-08-03

**Authors:** Bryan P. Koestner, William Hedgcock, Kameko Halfmann, Natalie L. Denburg

**Affiliations:** ^1^Department of Neurology, University of Iowa Carver College of MedicineIowa City, IA, USA; ^2^Department of Marketing, University of Iowa Tippie College of BusinessIowa City, IA, USA; ^3^Department of Psychology, Saint Norbert CollegeDe Pere, WI, USA

**Keywords:** MRI, decision making, aging, frontal lobe, fraud

## Abstract

Older adults are frequently the targets of scams and deception, with millions of individuals being affected each year in the United States alone. Previous research has shown that the ventromedial prefrontal cortex (vmPFC) may play a role in vulnerability to fraud. The current study examined brain activation patterns in relation to susceptibility to scams and fraud using functional magnetic resonance imaging (fMRI). Twenty-eight healthy, community-dwelling older adults were subdivided into groups of impaired and unimpaired decision makers as determined by their performance on the Iowa Gambling Task (IGT). While in the scanner, the participants viewed advertisements that were created directly from cases deemed deceptive by the Federal Trade Commission (FTC). We then obtained behavioral measures involving comprehension of claims and purchase intention of the product in each advertisement. Contrasts show brain activity in the vmPFC was less correlated with purchase intention in impaired vs. unimpaired older adult decision makers. Our results have important implications for both future research and recognizing the possible causes of fraud susceptibility among older adults.

## Introduction

Deceiving older adults is not a new problem, but it is one that has been growing in prevalence. To illustrate, financial abuse of elders aged 65 years and older has risen from 8% in 1950 to an astounding 20% in 2010 (Kemp and Liao, 2006; Infogroup/ORC, 2010). These recent statistics likely underestimate the extent of the problem, with only one in 25 cases being reported (Wasik, 2000). Moreover, these numbers do not take into account the devastation that fraud can have on older adults and their families, often wiping out entire savings in a single action, not to mention the psychological distress that ensues.

Recent evidence has shown that the ventromedial prefrontal cortex (vmPFC) may play a role in vulnerability to fraud. A study conducted by Asp et al. (2012) examined credulity, defined as a willingness to believe something even with minimal evidence, using real-world advertisements (adapted from Denburg et al., 2007) and a focal lesion approach. There were three study groups: patients with damage to the vmPFC; patients with damage to areas outside of the PFC; and a group of non-neurological, healthy adults. Results showed that those patients with lesions in the vmPFC were more likely to believe faulty claims and more likely to state that they wished to purchase products associated with deceptive advertising, as compared to both patients with lesions outside the PFC and the comparison sample of healthy adults.

The Asp et al. (2012) study was conducted in a patient population with known structural damage to the brain, but many healthy older adults succumb to the same troubling behavioral findings found in that study, such as believing deceptive claims or buying falsely advertised products (Denburg et al., 2005, 2007) examined decision making among healthy, community-dwelling older adults to see if individuals were susceptible to being scammed. The participants were first tested using the Iowa Gambling Task (IGT), which assesses decision making and has been shown to differentiate between people with intact and impaired vmPFC function (Bechara et al., 2000b; Denburg et al., 2007). In this test, each participant selects from four different decks of cards. Two of the decks are “Good” decks, in that the end result is a net gain in money, while two of the decks are “Bad” decks that result in a net loss of money. From their IGT performance, older participants were divided into three groups: unimpaired (selected predominately from the “Good” decks), impaired (selected predominately from the “Bad” decks), and borderline (did not perform differently than chance and thus was not studied further). A final group, composed of healthy young adults, aged 26–55 years old, was also administered the IGT and all selected predominately from the “Good” decks. Next, the unimpaired, impaired, and young adult groups were shown various advertisements that were either deceptive or non-deceptive in nature. Denburg et al. (2007) found that, when viewing *non-deceptive* advertisements, there were no group differences with regard to comprehension of claims and purchase intention of the product. However, this pattern changed markedly when viewing *deceptive* advertisements. The impaired decision makers were significantly more likely to endorse that they would purchase the product that was deceptively advertised, and also significantly more likely to miscomprehend the advertisements’ claim, as compared to both the unimpaired and young adult decision makers (who performed similarly).

A possible explanation for vmPFC vulnerability among older adults lies in a theory termed the frontal aging hypothesis (for a review see West, 1996). It states that the PFC is one of the first structures to undergo age-related decline. Support for this theory comes from a variety of sources, including brain, cognitive, and behavioral data. To illustrate, Raz et al. (1997) provided support for this theory in a cross-sectional study that examined brain volume in adults ranging in age from 18 to 77. Results revealed that the greatest age-related changes occurred in the PFC, with an average rate of volumetric decline of 4.9% per decade. Other studies have come to similar conclusions (e.g., Salat et al., 2004). There is also decline in cognitive functions related to the PFC, including working memory (Köstering et al., 2016), attention/distraction (Andrés et al., 2006), and the formerly mentioned decision making (Denburg et al., 2007).

Taken together, previous research has painted a picture for a plausible connection between healthy aging, decision making, and compromised integrity of the vmPFC, but research showing a direct link is scarce. The current research was conducted to examine whether an explicit connection between susceptibility to scams and abnormal vmPFC activity exists among older adults. To this end, we utilized functional neuroimaging to study decision making and brain activation in an older adult population. We hypothesized that the poor decision making shown in some normal, healthy older adults can be attributed to neurobiological decline in the function of the vmPFC. To test this, we measured the blood oxygen level-dependent (BOLD) signal in older adults using functional magnetic resonance imaging (fMRI) while they were presented deceptive and non-deceptive advertisements. We predicted that vmPFC activity in IGT-impaired older adults would be less correlated with comprehension and purchase intent than in IGT-unimpaired older adults.

## Materials and Methods

### Participants

Thirty-two participants were initially recruited from a registry compiled at the Department of Neurology, University of Iowa Hospitals and Clinics. This registry was comprised of healthy, community-dwelling older adults. Exclusion factors included head injury with extended loss of consciousness, stroke, Type 1 diabetes, neurosurgery, seizure disorder, demyelinating disorder, uncontrolled medical condition, significant vision/hearing loss, substance abuse, psychiatric illness necessitating inpatient treatment, and/or self-reported depression or anxiety exceeding mild severity. Four participants were ultimately excluded from the current study: two participants had no change in their purchase intention which made it impossible to estimate brain activity related to purchase intent, while data recording problems made it impossible to analyze two additional participants. Thus, the total number of participants in the current study was 28 (aged 62–88 years [*M* = 76.8, *SD* = 7.3]; 54% female). All participants gave written informed consent in accordance with the University of Iowa’s Institutional Review Board policy.

### Data Collected Prior to the fMRI Study

#### Neuropsychological Battery

Each of the 28 participants underwent extensive neuropsychological testing to ensure they were cognitively intact. All major domains of cognition were assessed, including premorbid intellect (Wide Range Achievement Test (WRAT) Reading subtest; Wilkinson, 1993; Wilkinson and Robertson, 2006), current intellect (Wechsler Abbreviated Scale of Intelligence, WASI; Wechsler, 1999), visual attention/retention (Benton Visual Retention Test, BVRT; Sivan, 1992), working memory (Wechsler Working Memory Index, WMI; Wechsler, 1997), visual anterograde memory (Rey-Osterrieth Complex Figure (ROCF) Delayed Memory; Osterrieth, 1944), verbal anterograde memory (Rey Auditory Verbal Learning Test (RAVLT) 30 min delay; Corwin and Bylsma, 1993a,b; Rey, 1941; Osterrieth, 1944), language (Controlled Oral Word Association Test, COWA; Benton and Hamsher, 1989), visuospatial skills (ROCF Copy; Osterrieth, 1944), mood (Beck Depression Inventory, BDI; Beck, 1987; Beck et al., 1996), and mental status screening (Mini Mental State Examination, MMSE; Folstein et al., 1975, 2001).

#### Iowa Gambling Task

The IGT is a computerized laboratory program that has been used in numerous studies for discerning complex decision making abilities (Bechara et al., 2000a; Ernst et al., 2002). The participant is presented with four different decks and is told that the goal of the game is to maximize profit. One card is picked from the top of a deck of their choice, and each card picked either represents a reward (monetary gain) or a reward followed by a punishment (monetary loss). Two of the decks are considered advantageous or “Good” decks (A and B), in the sense that they offer a small, immediate reward with lower long-term punishment. By contrast, the remaining two decks are considered disadvantageous or “Bad” decks (C and D), in the sense that they offer a large, immediate reward with greater long-term punishment. Thus, over time, picking from the “Good” decks will result in an accumulation of money while picking from the “Bad” decks will result in a loss of money. The participant is not told how many trials they will encounter (there are 100) nor are they told about the reward/punishment schedules. To quantify performance on the IGT, the number of selections from the “Good” decks were subtracted from the number of selections from the “Bad” deck to obtain an overall assessment of decision making ability: [(C + D) − (A + B)].

### fMRI Study

#### Stimuli and Procedure

When the participant arrived at the lab, they were first consented and asked general fMRI safety questions. The participant was then escorted to the fMRI room, checked for metal objects, and situated in the fMRI scanner.

The participants were presented with six advertisements while in the scanner, each of which were based on actual consumer product advertisements that were considered deceptive by the Federal Trade Commission (FTC; Federal Trade Commission, 2015). These advertisements were chosen and created with the desire to emulate stimuli that the general U.S. population (including older adults) see every day, and thus allow for a strong assessment of real-world consumer decision making. Two versions of each of the six advertisements were created: a deceptive version and a non-deceptive version. The deceptive versions were unchanged from the original FTC complaint, and thus either had false or omitted information that was vital for obtaining an accurate understanding of the product. By contrast, the non-deceptive versions were manipulated to include either corrected information or previously omitted information, and thus formed an accurate picture of the product being advertised. Although the advertisements were thematically the same for each participant^1^, whether they saw the deceptive or non-deceptive version was manipulated based on which of four conditions the participant was randomly assigned.

**Figure 1:**
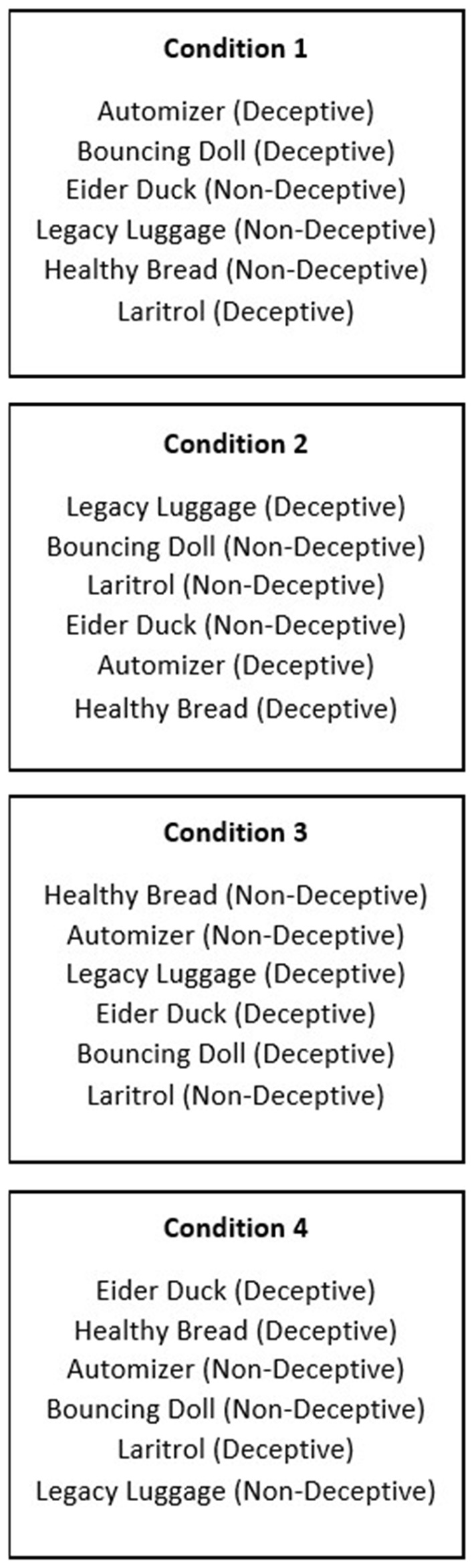
**Conditions, including which deceptive and non-deceptive versions were used**.

At the beginning of the experiment, the following instructions were provided on the screen: “You will see print advertisements on the following pages. Please read and review these advertisements. You will not have to answer any questions at this time, but we will ask you to evaluate the advertisements after you have left the scanner.” During the fMRI scan, each participant saw three deceptive and three non-deceptive advertisements. Which three advertisements were deceptive or non-deceptive was manipulated in each of four conditions, as well as the order in which the stimuli were presented (see Figure 1). Each advertisement was displayed for 20 s, with a 1.5 s fixation period between advertisements.

After the participant completed the fMRI portion of the experiment, they were escorted to a quiet room to complete a questionnaire concerning the advertisements they had just viewed. The questionnaire included two types of consumer questions: comprehension of claims and purchase intention. Comprehension of claims asked “What do you believe to be true about this product?”, and was completed on a 7-point non-numbered scale. For example, for the diet pill, one end of the scale stated “This product will absorb fat from the food I eat” (which is not true), while the other end read “This product will NOT absorb fat from the foods I eat” (which is true). The scale was then converted into corresponding numbers ranging from 1 (low comprehension) to 7 (high comprehension). Purchase intention questions were completed with a 5-point Likert scale, with one being “Likely” to purchase, 3 being “Neutral”, and five being “Unlikely” to purchase.

After the participant completed the questionnaire, they were asked if they had any questions or concerns about the study before being released. They were compensated by mail a couple of weeks later.

#### Scan Parameters

MRI scans were obtained using a standard 12-channel head array on a Siemens 3T TIM Trio. Patients lied supine in the bed with pillows used to help minimize distortion caused by head movement. A high-resolution full brain 3D MPRAGE sequence was collected for each participant in the transversal plane (256 × 256 × 256 mm T1-weighted, voxel size = 1 mm^3^, TI = 900 ms, TR = 2530 ms, TE = 3.04 ms, Flip angle = 10°, slice thickness = 1.0 mm, scan time = 10 min 48 s). During the advertising task, BOLD signal was measured with a T2* weighted echo-planar imaging (EPI) sequence. Standard scan parameters were used (TR = 2000, TE = 27, flip angle = 70°, FOV = 220 mm × 220 mm, in plane resolution = 64 × 64 pixels, slice thickness = 2.5 mm, 1.0 mm gap, voxel size = 3.4 × 3.4 × 2.5 mm). Thirty-three interlaced slices were acquired in an oblique orientation angled relative to the anterior commissure/posterior commissure line to help minimize PFC susceptibility problems. The first two volumes of each participant’s EPI scans were discarded to reduce problems with saturation effects.

### fMRI Processing and Analysis

fMRI analyses were performed using BrainVoyager QX 2.8 (Goebel et al., 2006). Anatomical data were corrected for image intensity inhomogeneities and transformed into Talairach space (Talairach and Tournoux, 1988) using the cubic spline interpolation method for initial ACPC transformation, then a sinc interpolation method for final Talairach transformation. All participants’ scans were manually inspected for poor registration. Functional data were pre-processed using standard steps including slice time correction using cubic spline interpolation and head motion in six directions using trilinear/sinc interpolation. We applied high-pass temporal filtering using Fast Fourier Transform to remove low-frequency drifts and spatially smoothed images using an 8 mm full-width-half-maximum Gaussian kernel. Functional images were registered to each individual’s high-resolution structural image which was collected in the same session. All statistical analyses were performed on a participant-averaged brain in standard Talairach space.

Statistical analyses were performed in a two-stage approach. In the first stage, we estimated a general linear model (GLM) with each part of the decision phase convolved using a two-gamma hemodynamic response function corrected for temporal serial correlation using an AR(2) model. The predictors included Fixation, Instructions, Ads, and a parametric regressor for behavior (depending on the analysis, this was either Comprehension or Purchase Intent) which was the mean-centered value for each individual’s response to each advertisement in the post-scanning questionnaire. We also included the six head motion parameters to each individuals first level GLM as nuisance variables. Fixation was treated as the baseline.

In the second stage, we used a multi-subject random-effects model with separate subject predictors. We applied a percent signal transformation of each value with respect to the voxel time course mean value. We created separate statistical maps for each participant and contrasted whole-brain BOLD activity for the unimpaired and impaired groups with a focus on comprehension- and purchase intent-correlated brain activity while viewing advertisements. These results were thresholded with a cluster-corrected significance level of *α* < 0.05, according to a three-dimensional extension of a randomization procedure first described by Forman et al. (1995). To achieve this, we first set the voxel-level threshold to *p* < 0.005 (one-tailed; uncorrected). Using the Cluster Threshold plugin in BrainVoyager and 5000 Monte Carlo stimulations, we then calculated cluster-level false positive rates.

## Results

### Demographic and Neuropsychological Data

Demographic and neuropsychological data are provided in Table 1. Overall, participants demonstrated average to high average intellectual functioning and normal cognitive performances for age and level of education (for assorted normative data, see Lezak et al., 2012). The scores on the MMSE were also near ceiling level. As expected for the IGT, a 2 × 5 repeated-measures ANOVA using participant group (impaired vs. unimpaired) as a between-subjects factor and trial block (Blocks 1–5) as a within-subjects factor yielded a significant two-way interaction (*F*_(4,104)_ = 4.95, *p* = 0.003)^2^, demonstrating the superior IGT performance of the unimpaired group vs. the impaired group. Importantly, when contrasting impaired vs. unimpaired participants, the demographic and neuropsychological data did not differ reliably (all *p*s > 0.05).

**Table 1:** **Means (SDs) and *p*-values for demographic and neuropsychological data**.

Measure	Unimpaired decision makers	Impaired decision makers	*p*-value
*N*	15	13	–
Age	76.9 (7.49)	76.6 (7.41)	0.911
Education	16.3 (2.89)	15.4 (2.79)	0.420
Sex	53% female	54% female	–
Handedness	14 RH, 1 LH	12 RH, 1 ambi	–
WRAT-Reading	111.9 (7.93)	108.1 (7.32)	0.203
WASI (FSIQ)	115.5 (33.4)	115.5 (12.9)	0.994
BVRT-Errors	3.33 (1.50)	3.46 (2.57)	0.871
WMI	112.0 (12.5)	112.4 (14.5)	0.941
ReyO delay	19.2 (5.31)	15.4 (5.90)	0.086
AVLT 30 min delay	10.7 (3.22)	9.08 (2.25)	0.148
BDI	3.87 (2.80)	5.15 (5.16)	0.411
COWA	47.4 (10.8)	39.4 (12.9)	0.86
ReyO copy	33.4 (2.69)	31.5 (3.60)	0.130
MMSE	29.5 (0.640)	29.2 (0.899)	0.294
IGT score	41.5 (18.6)	−27.8 (23.0)	0.000*

Note: Demographic and neuropsychological means, standard deviations, and *p*-values for unimpaired and impaired decision makers. RH, right handed; LH, left handed; ambi, ambidextrous; WRAT, Wide Range Achievement Test Reading Composite Score; WASI FSIQ, Wechsler Abbreviated Scale of Intelligence Full Scale Intelligence Quotient; BVRT-Errors, Benton Visual Retention Test (Errors made); WMI, Wechsler Working Memory Index; ReyO, Rey Osterrieth Complex Figure; AVLT 30 min Delay, Rey Auditory Verbal Learning Test 30 min Delay; BDI, Beck Depression Inventory; COWA, Controlled Oral Word Association Test; MMSE, Mini-Mental State Examination; IGT, Iowa Gambling Task. *Significant *p*-value.

### Behavioral Results

Our behavioral variables were comprehension of claims and purchase intention. Both variables were analyzed using two-tailed independent samples *t*-tests, as both fulfiled the assumptions of parametric *t*-tests, which includes approximate normality of the samples, equality of variances, and independence of the samples. The mean total comprehension score for the unimpaired group was 4.61 (*SD* = 1.07) and for the impaired group was 4.46 (*SD* = 0.86), with no significant difference found between the groups (*t* = 0.40, *p* = 0.69). The mean total purchase intention score for the unimpaired group was 3.91 (*SD* = 0.56) and for the impaired group was 3.95 (*SD* = 0.43), with no significant difference found between the groups (*t* = −0.20, *p* = 0.85). Thus, in terms of consumer behavior, the unimpaired and impaired decision makers had no statistically significant difference in comprehension of claims or purchase intention for the advertisements they saw.

Investigation of two-tailed Pearson’s correlations between the IGT, comprehension of claims, and purchase intention variables indicated relative independence of the variables. That is, the correlations between these variables were near zero: IGT and comprehension of claims, *r* = 0.102, *p* = 0.604; IGT and purchase intention, *r* = −0.036, *p* = 0.857; and comprehension of claims and purchase intention, *r* = −0.045, *p* = 0.821.

### fMRI BOLD Results

Our fMRI analyses focused on the BOLD activity obtained while participants were viewing the advertisements. We compared unimpaired and impaired decision makers to see whether brain activity during processing of the advertisements was differently correlated with our behaviors of interest. To do this, we used the parametric regressors described above. These regressors allowed us to disentangle comprehension- and purchase-related activity from other processes that occurred during advertisement viewing (e.g., visual processing, working memory). These regressors were based on each individual’s response to the post-scanning questionnaire for each product. While our hypotheses were focused on the vmPFC, we performed all analyses at the whole-brain level.

We first examined the parametric regressor for comprehension. Cluster thresholding for this contrast between unimpaired and impaired decision makers resulted in a minimum cluster size of 809 mm^3^. No volumes of interests (VOI) surpassed this or less conservative thresholds.

**Figure 2:**
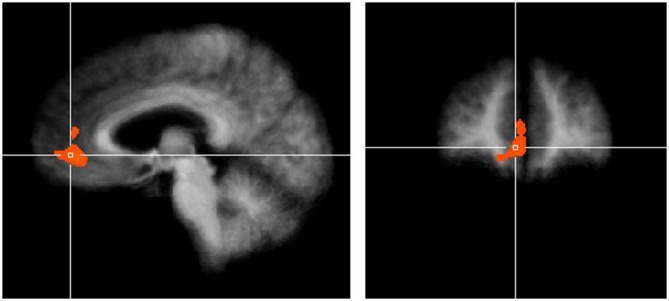
**Purchase intention-related brain activity in the ventromedial prefrontal cortex (vmPFC) differed between unimpaired and impaired participants.** Result shown is cluster corrected threshold of *α* = 0.05. Crosshairs are located at *x* = 5, *y* = 45, *z* = 0. Cell means for the volumes of interests (VOI) were 0.615 for unimpaired and −0.591 for impaired participants.

**Figure 3:**
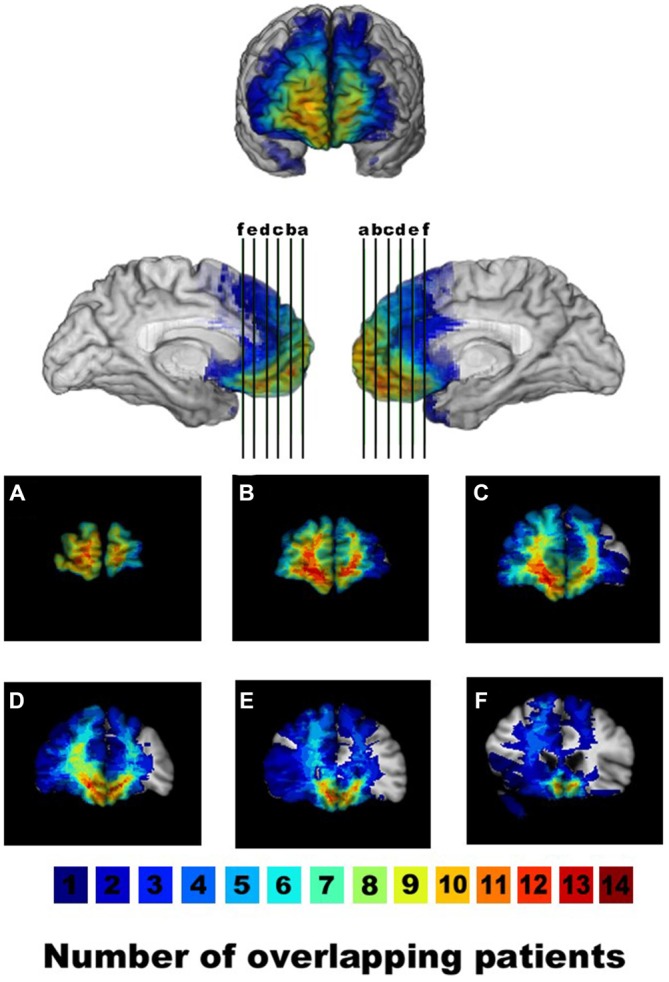
**Lesion overlap of patients with ventromedial prefrontal cortex lesions displayed in anterior/mesial views and coronal slices (A–F, with the right hemisphere on the left in the coronal views).** The color bar indicates the number of overlapping lesions per voxel. Reprinted with permission from Asp et al. (2012).

Next we examined the parametric regressor for purchase intention. Cluster thresholding for this contrast between unimpaired and impaired decision makers resulted in a minimum cluster size of 1064 mm^3^. One VOI in the vmPFC surpassed this thresholding. The cell means for the VOI in the vmPFC were 0.615 for the unimpaired participants and −0.591 for the impaired participants. Consistent with our hypotheses, brain activity in the vmPFC was less correlated with purchase intention in impaired vs. unimpaired older adult decision makers, as shown in Figure 2. Further comparison shows that the area of significant difference in our analysis overlaps with the area of maximal brain lesion overlap in the Asp et al. (2012) study, as shown in Figure 3. Additional details for these results can be found in Table 2.

**Table 2:** **Whole-brain results contrasting impaired and unimpaired decision makers**.

Region	BA	Peak *x*	Peak *y*	Peak *z*	mm^3^	Mean *x*	Mean *y*	Mean *z*
Anterior cingulate	32	3	41	−2	1656	5.41	44.59	0.28

## Discussion

Our research suggests that the cognitive process supported by the vmPFC may play a role in older adults’ susceptibility to scams. Specifically, brain activation in the vmPFC was less correlated with purchase intention in participants who were defined as impaired vs. unimpaired based on their performance on the IGT. These results help confirm the earlier findings of Denburg et al. (2005), Denburg and Harshman (2010) and Asp et al. (2012), which suggested that age-related changes to the brain that are detected using the IGT affect both susceptibility to fraud and brain activity in the vmPFC.

Although some of the hypothesized brain activity differences were supported, we found that there was no statistically significant difference between the unimpaired and impaired groups on our behavioral variables. That is, the groups did not significantly differ in their reported comprehension of claims or purchase intention. There are a number of possible explanations for this variance. The first is a statistical explanation—the small number of stimuli may have meant we did not have the statistical power necessary to measure subtle differences between these two groups. The second is a cognitive explanation—these two groups of older adults ended up with the same behavioral evaluations of the advertisements but came to these conclusions using different neurocognitive mechanisms. The compensation-related utilization of neural circuits hypothesis (CRUNCH; Reuter-Lorenz and Cappell, 2008) predicts our exact pattern of results—similar behavioral performance but different brain activation—when the level of task difficulty is low. This seemingly inconsistent pattern of results occurs when older adults maintain performance by recruiting additional resources when tasks are relatively easy, but performance drops off when task difficulty increases. Future research could examine whether the performance of our impaired population decreases when task difficulty is increased. Another approach for future studies could involve altering how participants’ decision making is initially assessed. In the present study, we used the IGT to determine baseline decision making, but an alternative method could involve using an advertising task, similar to the one used in the fMRI. This would more directly assess participants’ susceptibility to deceptive advertisement and could improve the odds of seeing behavioral differences between the groups while they are in the scanner.

Several limitations must be acknowledged. First, each participant spent a relatively brief amount of time in the scanner. The participants viewed six advertisements for 20 s each. This helped reduce subject-related issues such as movement and boredom that can occur with longer studies. However, it also resulted in lower statistical power and necessarily required a relatively simple experimental design. In future studies, we would like to see this time increased by adding additional advertisements to the protocol. Second, our study, like most fMRI studies (Button et al., 2013), has low statistical power. This means that while our sample size is reasonably large for an fMRI study and is consistent with prior work (Balardin et al., 2015; Luis et al., 2015; Ramanoël et al., 2015), we likely did not detect any true effects and may have overestimated the effect sizes. Care should thus be taken when interpreting null results and magnitude of effects. Consideration of experimental designs that could promote statistical power (e.g., within subjects designs) would be fruitful future directions.

There are real-world applications for our research findings. First, we now have direct evidence that the vmPFC is associated with older adults’ susceptibility to scams. Through pharmacotherapy, we may be able to target this brain area in the future as a way for older adults to avoid faulty consumer decision making. Evidence has been shown that poor decision-making can be linked to abnormalities in both serotonin and dopamine in the brain, specifically in the PFC region (for a relevant review, see Rogers, 2011). Future studies could look into the effects that drugs like selective serotonin reuptake inhibitors (SSRIs) would have on decision making in older adults. Second, as neuroimaging becomes more affordable, it may be possible to preventatively identify or even “diagnose” older adults who are more likely to be susceptible to fraud. Until then, laboratory tasks that specifically target vmPFC function, such as the IGT, could be used as a diagnostic test to help identify and help potentially vulnerable individuals, such as by implementing safety nets to protect their loved one.

In conclusion, our research suggests that abnormal processing in the vmPFC may contribute to susceptibility to scams and fraud in some older adults. Future research will determine if these changes are due to normal aging, and if so, how to prevent such age-related neurodegeneration or find ways to protect older adults who are susceptible. Far too many of our older adults are falling prey to scams and losing everything they have worked so hard for—pursuing further research and ideas on how to protect them should be of the upmost importance.

## Author Contributions

BPK, WH, and NLD designed the research; BPK, WH, KH, and NLD performed the research; BPK, WH, KH, and NLD analyzed the data; and BPK, WH, KH, and NLD wrote the article.

## Funding

Funding for this research was supported by a DANA Foundation Program in Brain and Immuno-Imaging grant to NLD.

## Conflict of Interest Statement

The authors declare that the research was conducted in the absence of any commercial or financial relationships that could be construed as a potential conflict of interest.

## Footnotes

^1^The following illustrates the difference between the deceptive and non-deceptive advertisement for a diet pill. The deceptive advertisement states that studies have shown that the diet pill can eliminate a number of grams of fat directly from food that is eaten. The non-deceptive advertisement states that studies have shown that the pill, combined with diet and exercise, can help an individual lose body fat. The non-deceptive advertisement contains information that the FTC deems necessary to allow the consumer to be fully informed about the product as compared to the deceptive advertisement.

^2^The group (*F*_(1,26)_ = 83.11, *p* < 0.0001) and trial block (*F*_(4,104)_ = 3.75, *p* = 0.008) main effects were also significant.
